# Discovery and validation of the prognostic value of the lncRNAs encoding snoRNAs in patients with clear cell renal cell carcinoma

**DOI:** 10.18632/aging.102894

**Published:** 2020-03-03

**Authors:** Wuping Yang, Kenan Zhang, Lei Li, Kaifang Ma, Baoan Hong, Yanqing Gong, Kan Gong

**Affiliations:** 1 Department of Urology, Peking University First Hospital, Beijing 100034, P.R. China; 2 Hereditary Kidney Cancer Research Center, Peking University First Hospital, Beijing 100034, P.R. China; 3 Institute of Urology, Peking University, Beijing 100034, P.R. China; 4 National Urological Cancer Center, Beijing 100034, P.R. China

**Keywords:** ccRCC, SNHG3, SNHG15, prognosis, DNA methylation

## Abstract

Some lncRNAs can encode small nucleolar RNAs (snoRNAs), called small nucleolar RNA host genes (SNHGs), which have exerted certain predictive values for the prognosis of some cancer patients. In this study, using RNA-seq and survival data in TCGA-KIRC, we examined the expression profile of 20 SNHGs and explored their prognostic values in ccRCC. Results showed that SNHG1, GAS5, SNHG3-8, SNHG11, SNHG12, SNHG15-17, SNHG20, SNHG22 and SNHG25 were significantly upregulated in ccRCC tissues compared with adjacent normal tissues. After adjustment for confounding factors, the multivariate analysis confirmed that increased SNHG3 expression was independently associated with shorter OS, while increased SNHG15 expression was an independent predictor of shorter RFS. Using the methylation data, the methylation status of 2 CpG sites (cg07807470 and cg15161854) and 2 CpG sites (cg00953154 and cg16459265) were negatively correlated with SNHG3 and SNHG15 expression, respectively. Moreover, low methylation levels of the 4 CpG sites were significantly associated with shorter OS. Furthermore, we validated the expression patterns, methylation status and prognostic value of SNHG3 and SNHG15 using clinical ccRCC samples. Taken together, SNHG3 and SNHG15 might be valuable prognostic markers in ccRCC, and DNA hypomethylation might play an important role in elevated SNHG3 and SNHG15 transcription in ccRCC.

## INTRODUCTION

Renal cell carcinoma (RCC) is one of the most aggressive cancers of the urinary system, accounting for approximately 4% of adult malignancies [1]. The most common histologic subtype clear cell RCC (ccRCC) accounts for approximately 75-80% of RCC, and up to 92% of these cancers have VHL protein inactivation [2–4]. Mutations and inactivation of VHL lead to accumulation of HIF-α proteins and upregulation of HIF-α target genes, which has been considered as a key mechanism to promote the progression of ccRCC [5]. In spite of recent advances in the comprehensive treatments like surgical operation, molecular targeted therapy, chemotherapy and radiation treatment, the 5-year overall survival rate for metastatic ccRCC patients remains as low as 10% to 20% [6]. Therefore, it is imperative to identify new biomarkers and therapeutic targets for ccRCC.

With the development of high-throughput transcriptome analysis in recent years, it has been found that over 90% of the total mammalian genome can be transcribed but does not encode proteins [7]. As a new class of non-coding RNA (ncRNA), long ncRNAs (lncRNAs) longer than 200 nucleotides have been found to be aberrantly expressed in some diseases, particularly in cancer [8, 9]. Although lncRNAs are not involved in protein-coding, they can regulate gene expression at the level of chromatin modification, transcription and post-transcriptional processing [10]. In addition, emerging studies indicate that lncRNAs are differentially expressed in ccRCC and exert critical regulatory effects on a series of cellular processes, such as proliferation, apoptosis and metastasis [11, 12].

Some lncRNAs can encode small nucleolar RNAs (snoRNAs), called small nucleolar RNA host genes (SNHGs). snoRNAs have been considered to be one of the best characterized classes of non-coding RNAs (ncRNAs) with a wide variety of cellular functions, such as chemical RNA modifications (such as methylations and pseudouridylations), pre-RNA processing and alternative splicing control [13–15]. In addition, some snoRNAs have shown differential expression patterns in various human cancers, as well as the ability to affect cell transformation, tumorigenesis, and metastasis [16–18]. For instance, SNORA21 showed oncogenic properties in human colorectal cancer, and elevated SNORA21 served as an independent factor for predicting poor survival [19]; SNORA42 expression was an independent prognostic factor for overall survival and disease-free survival of colorectal cancer [20]. Thus, the SNHGs, as the host genes of snoRNAs, may have multiple regulatory effects on tumor cell processes and play crucial roles in cancer.

Consistent with the above researches, several recent studies have demonstrated that the SNHGs might be valuable prognostic markers in some cancers. GAS5 could inhibit the progression of colorectal cancer by interacting with and triggering YAP phosphorylation and degradation [21]; SNHG15 interacted with and stabilized transcription factor Slug and promoted colon cancer progression [22]; Higher SNHG20 expression was significantly associated with advanced tumor, lymph node and metastases (TNM) stage and tumor size, as well as poorer overall survival [23]. However, there has been relatively little research on the clinical roles of SNHGs in ccRCC specifically. In this study, using RNA-seq and survival data in the Cancer Genome Atlas (TCGA)-Kidney Renal Clear Cell Carcinoma (KIRC), we examined the expression profile of some SNHGs and explored their prognostic values in ccRCC, followed by validation in a certain number of paired clinical samples (adjacent normal renal tissue and ccRCC).

## RESULTS

### The identification of differentially expressed lncRNAs encoding snoRNAs in ccRCC

**Figure 1:**
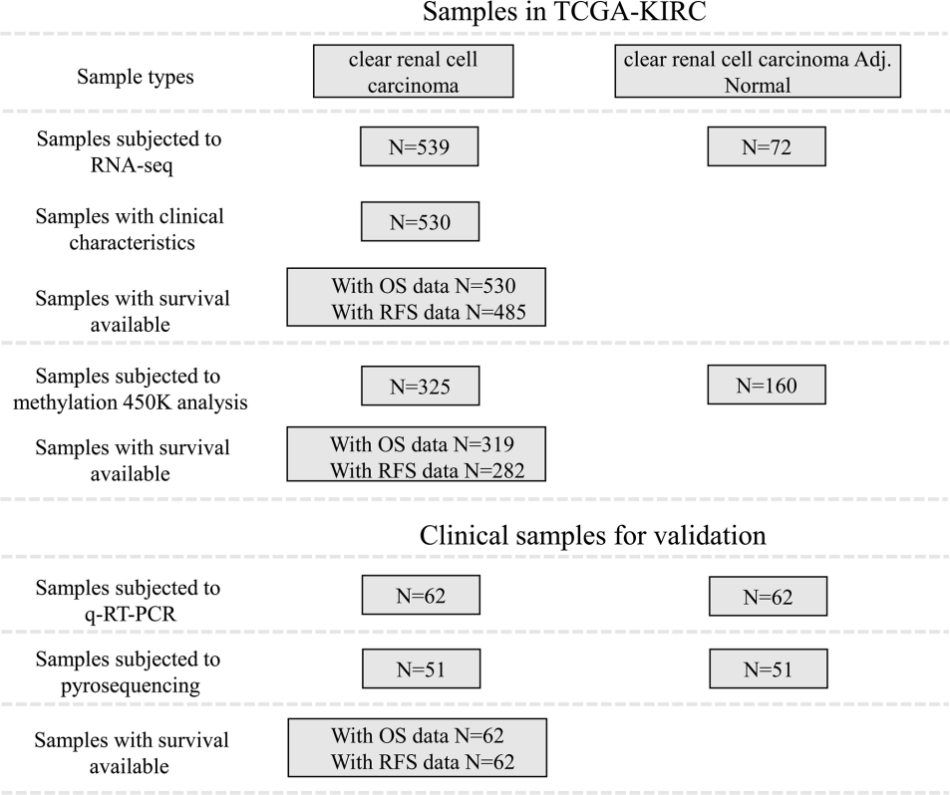
**Patient inclusion and data availability.**

**Figure 2:**
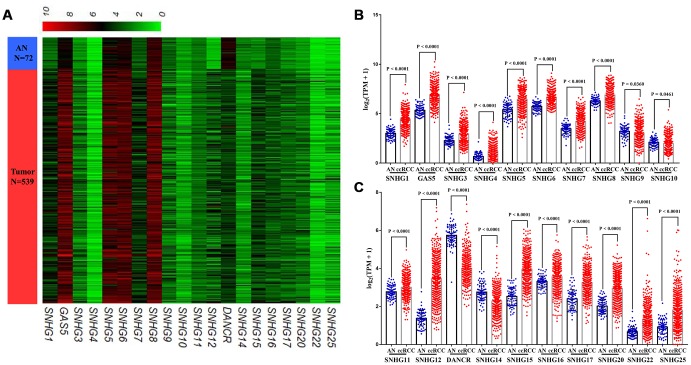
**Expression profiles of lncRNAs encoding snoRNA in ccRCC.** (**A**) Heatmap, (**B**, **C**) Plots chart showing the expression profile of correlation between SNHG1, GAS5, SNHG3-12, DANCR, SNHG14-17, SNHG20, SNHG22 and SNHG25 between ccRCC tissues and matched adjacent (adj.) normal (AN) tissues.

In TCGA-KIRC cohort, tumor tissues from 539 cases of ccRCC were subjected to RNA-seq study, among which 72 cases had matched adjacent normal tissues (Figure 1). Using RNA-seq data in TCGA-KIRC, we compared the expression of lncRNAs encoding snoRNAs between ccRCC tissues and the matched adjacent normal tissues. Results showed that compared with adjacent normal tissues, SNHG1, GAS5, SNHG3-8, SNHG11, SNHG12, SNHG15-17, SNHG20, SNHG22 and SNHG25 were significantly upregulated in ccRCC tissues, while SNHG9, SNHG10, DANCR and SNHG14 were remarkably downregulated in ccRCC tissues (Figure 2A–2C).

### Association between lncRNAs encoding snoRNAs and survival of ccRCC patients

**Figure 3:**
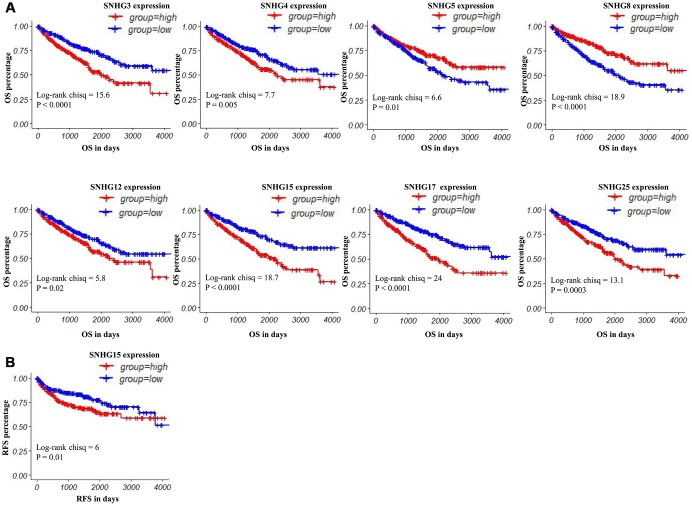
**Kaplan–Meier curves of OS and RFS in patients with ccRCC.** (**A**) Patients were grouped according to the median cutoff of SNHG3, SNHG4, SNHG5, SNHG8, SNHG12, SNHG15, SNHG17 and SNHG25 expression for OS detection. (**B**) Patients were grouped according to the median cutoff of SNHG15 expression for RFS detection.

To explore the prognostic values of lncRNAs encoding snoRNAs, we used the TCGA-Clinical data to analyze their relevances with the Overall Survival (OS) and Relapse-Free survival (RFS) among ccRCC patients by generating Kaplan–Meier survival curves (Figure 1), and the detailed clinical characteristics of patients with ccRCC in TCGA were shown in Table 1. Results of the log-rank test showed that the high expression groups of SNHG3, SNHG4, SNHG12, SNHG15, SNHG17 and SNHG25 had significantly shorter OS, while high SNHG5 and SNHG8 expression groups had remarkably longer OS (Figure 3A). Moreover, results of the log-rank test also showed that the high SNHG15 expression group had significantly shorter RFS than its low expression group (Figure 3B).

**Table 1:** The clinical characteristics of 530 ccRCC patients in TCGA.

**Clinicopathologic characteristics**		**n (%)**
		
Age		
	<60	245 (46.2)
	>=60	285 (53.8)
Overall Survival		
	Alive	373 (70.4)
	Dead	157 (29.6)
Gender		
	Male	344 (64.9)
	Female	186 (35.1)
Tumor stage		
	T1/T2	340 (64.2)
	T3/T4	190 (35.8)
Lymphatic invasion		
	N0	239 (45.1)
	N1	16 (3.0)
	NX	275 (51.9)
Metastasis		
	M0	440 (83.0)
	M1	80 (15.1)
	MX	10 (1.9)
Pathological stage		
	I/II	322 (61.1)
	III/IV	205 (38.9)
Historical grade		
	G1/G2	241 (45.7)
	G3/G4	286 (54.3)

In univariate analysis, advanced tumor stage/pathological stage/histological grade, metastasis, increased SNHG3/SNHG4 expression and decreased SNHG5/SNHG8 expression were potential risk factors of shorter OS. After adjustment for confounding factors, the multivariate analysis indicated that only advanced pathological stage, metastasis and increased SNHG3 expression were independently associated with shorter OS in ccRCC patients (Table 2). In terms of RFS, advanced tumor stage/pathological stage/histological grade, metastasis and increased SNHG15 expression were potential risk factors of shorter RFS. Moreover, the following multivariate analysis showed that advanced pathological stage/histological grade, metastasis and increased SNHG15 expression were independent predictors of shorter RFS in ccRCC patients (Table 3).

**Table 2:** Univariate and multivariate analysis of OS in patients with ccRCC.

**Parameters**	**Univariate analysis**				**Multivariate analysis**			
	**P**	**HR**	**95%CI**		**P**	**HR**	**95%CI**	
			**Lower**	**Upper**			**Lower**	**Upper**
**Age(Continuous)**	0.4410	1.1280	0.8306	1.5310				
**Gender**								
Male		1.0000						
Female	0.8190	1.0380	0.7555	1.4260				
**Tumor stage**								
T1/T2		1.0000						
T3/T4	**<0.0001**	3.2030	2.3470	4.3680	0.8367	0.9364	0.5014	1.7490
**Metastasis**								
No		1.0000						
yes	**<0.0001**	4.4130	3.2270	6.0350	**<0.0001**	2.6229	1.7469	3.9380
**Pathological stage**								
I/II		1.0000						
III/IV	**<0.0001**	3.8760	2.8010	5.3630	**0.0071**	2.6986	1.3103	5.5580
**Histological grade**								
G1/G2		1.0000						
G3/G4	**0.0236**	1.4390	1.0500	1.9720	0.0780	0.7172	0.4956	1.0380
**SNHG3**	**0.0008**	2.4240	2.0543	2.9474	**0.0362**	1.3401	1.0556	1.7010
**SNHG4**	**0.0178**	1.3300	1.0510	1.6830	0.4216	1.1586	0.8091	1.6590
**SNHG5**	**0.0011**	0.7783	0.6698	0.9044	0.0631	0.8366	0.6931	1.0100
**SNHG8**	**0.0146**	0.7908	0.6551	0.9547	0.6159	0.9400	0.7382	1.1970
SNHG12	0.2140	1.0980	0.9473	1.2730				
SNHG15	0.1050	1.1830	0.9655	1.4510				
SNHG17	0.1630	1.1810	0.9349	1.4910				
SNHG25	0.1590	1.1240	0.9550	1.3240				

**Table 3:** Univariate and multivariate analysis of RFS in patients with ccRCC.

**Parameters**	**Univariate analysis**				**Multivariate analysis**			
	**P**	**HR**	**95%CI**		**P**	**HR**	**95%CI**	
			**Lower**	**Upper**			**Lower**	**Upper**
**Age (Continuous)**	0.8050	1.0020	0.9864	1.0180				
**Gender**								
Male		1.0000						
Female	0.0680	0.6767	0.4449	1.0290				
**Tumor stage**								
T1/T2		1.0000						
T3/T4	**<0.0001**	4.5180	3.0690	6.6510	0.1967	0.6334	0.3167	1.2669
**Metastasis**								
No		1.0000						
yes	**<0.0001**	12.0300	8.0760	17.9100	**<0.0001**	4.6964	2.9147	7.5673
**Pathological stage**								
I/II		1.0000						
III/IV	**<0.0001**	6.8300	4.4650	10.4500	**0.0002**	4.8308	2.1181	11.0177
**Histological grade**								
G1/G2		1.0000						
G3/G4	**<0.0001**	3.3350	2.1480	5.1760	**0.0190**	1.7529	1.0966	2.8020
**SNHG15**	**0.0010**	1.5440	1.1920	2.0000	**0.0248**	1.3750	1.0412	1.8158

**Figure 4:**
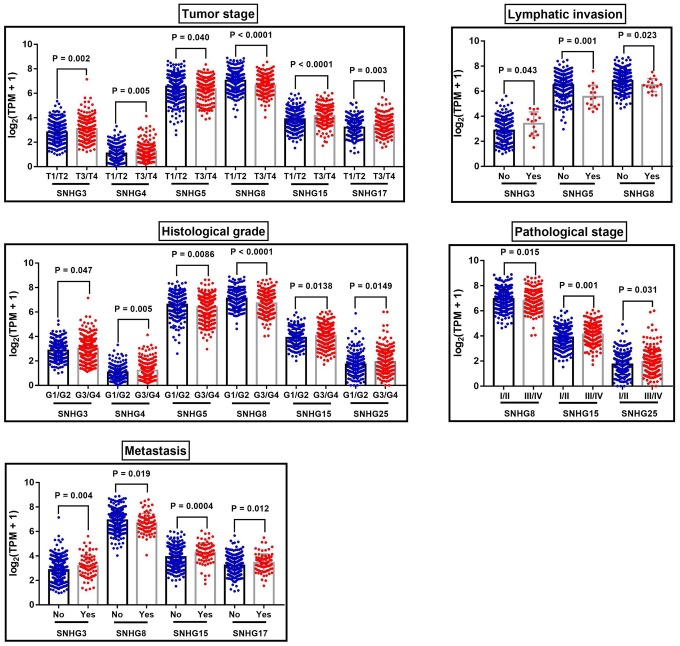
**The correlation between SNHGs expression and the clinical characteristics of ccRCC patients.** The correlation between SNHGs expression and tumor stage, lymphatic invasion, histological grade, pathological stage and metastasis.

In addition, these SNHGs expression were closely related to some clinical parameters of ccRCC, including tumor stage, lymphatic invasion, metastasis, pathological stage and histological grade (Table 4, Figure 4). Compared with early-stage (T1/T2) ccRCC, the expression levels of SNHG3, SNHG4, SNHG15 and SNHG17 were significantly higher in advanced-stage (T3/T4) ccRCC. Moreover, SNHG3, SNHG4 and SNHG15 were highly expressed in high histological grade ccRCC compared to low histological grade ccRCC. Furthermore, the expression levels of SNHG3 and SNHG15 in ccRCC with remote metastasis were remarkably higher.

**Table 4:** The correlation between SNHGs expression and the clinical characteristics of ccRCC patients in TCGA-KIRC.

**lncRNAs**	**Tumor stage**		**Lymphatic invasion**		**Metastasis**		**Pathological stage**		**Histological grade**	
	**T1/T2 vs.T3/T4**		**No vs.Yes**		**No vs.Yes**		**I/II vs.III/IV**		**G1/G2 vs.G3/G4**	
	**t**	**p**	**t**	**p**	**t**	**p**	**t**	**p**	**t**	**p**
**SNHG3**	**-3.181**	**0.002**	**-2.195**	**0.043**	**-2.971**	**0.004**	-1.390	0.165	**-1.994**	**0.047**
**SNHG4**	**-2.796**	**0.005**	-2.017	0.061	-1.679	0.096	-0.909	0.364	**-2.824**	**0.005**
**SNHG5**	**2.062**	**0.040**	3.943	0.001	1.258	0.211	1.105	0.270	**2.638**	**0.0086**
**SNHG8**	**5.690**	**<0.0001**	**2.462**	**0.023**	**2.389**	**0.019**	**2.453**	**0.015**	**5.891**	**<0.0001**
**SNHG12**	-1.532	0.127	-1.429	0.172	-1.839	0.070	0.347	0.729	-0.931	0.3522
**SNHG15**	**-3.943**	**<0.0001**	-0.180	0.859	**-3.658**	**0.0004**	**-3.506**	**0.001**	**-2.472**	**0.0138**
**SNHG17**	**-3.036**	**0.003**	-1.406	0.178	**-2.560**	**0.012**	-1.370	0.171	-1.559	0.1197
**SNHG25**	-1.556	0.121	-1.439	0.169	-1.922	0.057	**-2.162**	**0.031**	**-2.444**	**0.0149**

### SNHG3 and SNHG15 expression was regulated by DNA methylation

**Figure 5:**
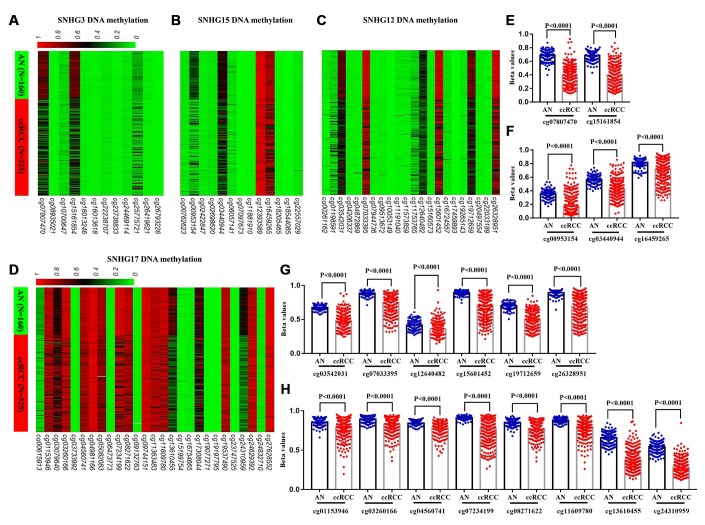
**Comparison of SNHG3, SNHG15, SNHG12 and SNHG17 DNA methylation in ccRCC and adjacent normal tissues.** (**A**–**D**) Heatmaps and (**E**–**H**) statistical comparison of the difference in methylation in CpG sites of SNHG3, SNHG15, SNHG12 and SNHG17 DNA, between 325 ccRCC and 160 adjacent normal tissues.

In TCGA-KIRC, 325 ccRCC tissues samples and 160 adjacent normal tissues samples were subjected to DNA methylation analysis simultaneously (Figure 1). Using the methylation data obtained from the Infinium Human Methylation450 BeadChip, we compared the methylation status of 12 CpG sites in SNHG3 DNA, 13 CpG sites in SNHG15 DNA, 22 CpG sites in SNHG12 DNA and 24 CpG sites in SNHG17 DNA (Table 5). The heatmap constructed on beta values of these CpG sites across all 485 samples was shown in Figure 5A–5D. The analysis results showed that compared with adjacent normal tissues, 2 sites of SNHG3 DNA, 3 sites of SNHG15 DNA, 6 sites of SNHG12 DNA and 8 sites of SNHG17 DNA were significantly hypomethylated in ccRCC (Figure 5E–5H).

**Table 5:** The detailed information of CpG sites of SNHG3, SNHG15, SNHG12 and SNHG17 DNA.

**Composite element REF**	**Chromosome**	**Start**	**End**	**CGI_Coordinate**
**SNHG3**				
cg07807470	chr1	28510838	28510839	CGI:chr1:28518141-28518781
cg08935021	chr1	28506029	28506030	CGI:chr1:28518141-28518781
cg10700647	chr1	28506126	28506127	CGI:chr1:28518141-28518781
cg15161854	chr1	28509627	28509628	CGI:chr1:28518141-28518781
cg16013246	chr1	28505956	28505957	CGI:chr1:28518141-28518781
cg16013618	chr1	28507076	28507077	CGI:chr1:28518141-28518781
cg22238707	chr1	28506552	28506553	CGI:chr1:28518141-28518781
cg23738833	chr1	28505869	28505870	CGI:chr1:28518141-28518781
cg24469114	chr1	28506476	28506477	CGI:chr1:28518141-28518781
cg25775721	chr1	28505362	28505363	CGI:chr1:28518141-28518781
cg26419621	chr1	28507275	28507276	CGI:chr1:28518141-28518781
cg26793226	chr1	28506061	28506062	CGI:chr1:28518141-28518781
**SNHG15**				
cg00762623	chr7	44986421	44986422	CGI:chr7:44986359-44987027
cg00953154	chr7	44986021	44986022	CGI:chr7:44986359-44987027
cg02422847	chr7	44986984	44986985	CGI:chr7:44986359-44987027
cg02698620	chr7	44986980	44986981	CGI:chr7:44986359-44987027
cg03440944	chr7	44983730	44983731	CGI:chr7:44986359-44987027
cg06057141	chr7	44986818	44986819	CGI:chr7:44986359-44987027
cg07097673	chr7	44986749	44986750	CGI:chr7:44986359-44987027
cg11881910	chr7	44986660	44986661	CGI:chr7:44986359-44987027
cg12393589	chr7	44988044	44988045	CGI:chr7:44986359-44987027
cg16459265	chr7	44985481	44985482	CGI:chr7:44986359-44987027
cg18205465	chr7	44986944	44986945	CGI:chr7:44986359-44987027
cg18544085	chr7	44986751	44986752	CGI:chr7:44986359-44987027
cg22557029	chr7	44986812	44986813	CGI:chr7:44986359-44987027
**SNHG12**				
cg00261162	chr1	28580966	28580967	CGI:chr1:28581557-28582287
cg01198591	chr1	28582493	28582494	CGI:chr1:28581557-28582287
cg03542031	chr1	28579698	28579699	CGI:chr1:28581557-28582287
cg04206337	chr1	28581041	28581042	CGI:chr1:28581557-28582287
cg04872869	chr1	28582125	28582126	CGI:chr1:28581557-28582287
cg07033395	chr1	28580026	28580027	CGI:chr1:28581557-28582287
cg07944736	chr1	28581714	28581715	CGI:chr1:28581557-28582287
cg09513672	chr1	28582258	28582259	CGI:chr1:28581557-28582287
cg10053149	chr1	28581997	28581998	CGI:chr1:28581557-28582287
cg11191040	chr1	28581122	28581123	CGI:chr1:28581557-28582287
cg11573859	chr1	28581227	28581228	CGI:chr1:28581557-28582287
cg11753765	chr1	28582446	28582447	CGI:chr1:28581557-28582287
cg12640482	chr1	28579061	28579062	CGI:chr1:28581557-28582287
cg15160573	chr1	28582130	28582131	CGI:chr1:28581557-28582287
cg15601452	chr1	28579870	28579871	CGI:chr1:28581557-28582287
cg16724557	chr1	28581391	28581392	CGI:chr1:28581557-28582287
cg17459893	chr1	28582020	28582021	CGI:chr1:28581557-28582287
cg19265143	chr1	28581125	28581126	CGI:chr1:28581557-28582287
cg19712659	chr1	28579844	28579845	CGI:chr1:28581557-28582287
cg20497554	chr1	28581859	28581860	CGI:chr1:28581557-28582287
cg22033189	chr1	28582039	28582040	CGI:chr1:28581557-28582287
cg26328951	chr1	28580002	28580003	CGI:chr1:28581557-28582287
**SNHG17**				
cg00615913	chr20	38434453	38434454	CGI:chr20:38434882-38435463
cg01153946	chr20	38433978	38433979	CGI:chr20:38434882-38435463
cg03079640	chr20	38435631	38435632	CGI:chr20:38434882-38435463
cg03260166	chr20	38431074	38431075	CGI:chr20:38434882-38435463
cg04560741	chr20	38427432	38427433	CGI:chr20:38434882-38435463
cg04981166	chr20	38428775	38428776	CGI:chr20:38434882-38435463
cg06473773	chr20	38435086	38435087	CGI:chr20:38434882-38435463
cg07234199	chr20	38431182	38431183	CGI:chr20:38434882-38435463
cg08271622	chr20	38425321	38425322	CGI:chr20:38434882-38435463
cg09744151	chr20	38429772	38429773	CGI:chr20:38434882-38435463
cg11363483	chr20	38429853	38429854	CGI:chr20:38434882-38435463
cg11609780	chr20	38431293	38431294	CGI:chr20:38434882-38435463
cg13610455	chr20	38426257	38426258	CGI:chr20:38434882-38435463
cg15199754	chr20	38435363	38435364	CGI:chr20:38434882-38435463
cg16754665	chr20	38435462	38435463	CGI:chr20:38434882-38435463
cg17308044	chr20	38435758	38435759	CGI:chr20:38434882-38435463
cg19077271	chr20	38435352	38435353	CGI:chr20:38434882-38435463
cg19197795	chr20	38435356	38435357	CGI:chr20:38434882-38435463
cg19537490	chr20	38428539	38428540	CGI:chr20:38434882-38435463
cg23747525	chr20	38435446	38435447	CGI:chr20:38434882-38435463
cg24310959	chr20	38426237	38426238	CGI:chr20:38434882-38435463
cg24629392	chr20	38428723	38428724	CGI:chr20:38434882-38435463
cg24832710	chr20	38435451	38435452	CGI:chr20:38434882-38435463
cg27628552	chr20	38435861	38435862	CGI:chr20:38434882-38435463

**Figure 6:**
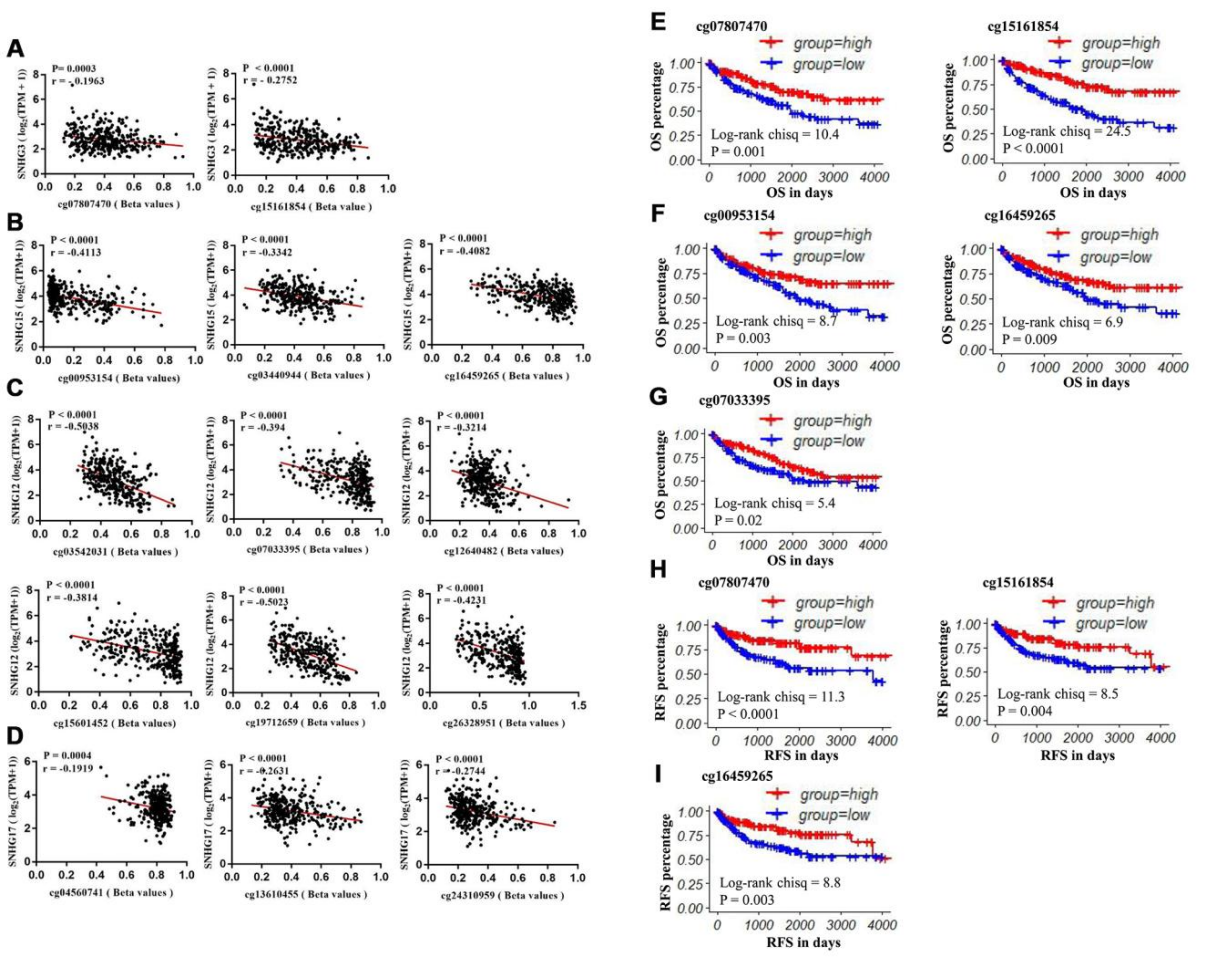
**The prognostic value of CpG sites that are negatively correlated with SNHG3, SNHG15, SNHG12 and SNHG17 expression.** (**A**) The correlation between SNHG3 expression and the methylation status of cg07807470 and cg15161854. (**B**) The correlation between SNHG15 expression and the methylation status of cg00953154, cg03440944 and cg16459265. (**C**) The correlation between SNHG12 expression and the methylation status of cg03542031, cg07033395, cg12640482, cg15601452, cg19712659 and cg26328951. (**D**) The correlation between SNHG12 expression and the methylation status of cg04560741, cg13610455 and cg24310959. (**E**–**G**) Patients were grouped according to the median cutoff of cg07807470, cg15161854, cg00953154, cg16459265 and cg0703395 methylation status for OS detection. (**H**–**I**) Patients were grouped according to the median cutoff of cg07807470, cg15161854 and cg16459265 methylation status for RFS detection.

To further explore the potential regulatory effect of DNA methylation on SNHG3, SNHG15, SNHG12 and SNHG17 expression, we analyzed the correlation between the 4 lncRNAs expression and the methylation status of their CpG sites. Correlation analysis results showed that the expressions of SNHG3, SNHG15, SNHG12 and SNHG17 were negatively associated with the methylation levels of 2 sites (cg07807470 r=-0.1963, cg15161854 r=-0.2752), 3 sites (cg00953154 r=-0.4113, cg03440944 r=-0.3342, cg16459265 r=-0.4082), 6 sites (cg03542031 r=-0.5038, cg07033395 r=-0.394, cg12640482 r=-0.3214, cg15601452 r=-0.3814, cg19712659 r=-0.5023, cg26328951 r=-0.4231) and 3 sites (cg04560741 r=-0.1919, cg13610455 r=-0.2631, cg24310959 r=-0.2744), respectively (Figure 6A–6D). Moreover, results of the log-rank test showed that low methylation levels of cg07807470 (SNHG3), cg15161854 (SNHG3), cg00953154 (SNHG15), cg16459265 (SNHG15) and cg07033395 (SNHG12) were significantly associated with the shorter OS of ccRCC patients (Figure 6E–6G). Furthermore, low methylation levels of cg07807470 (SNHG3), cg15161854 (SNHG3) and cg16459265 (SNHG15) were also related to the shorter RFS (Figure 6H, 6I). Taken together, these results indicated that the expressions of SNHG3 and SNHG15 were more likely to be modulated by DNA methylation in ccRCC.

### The validation of the expression patterns and the DNA methylation status of SNHG3 and SNHG15 based on clinical samples

**Figure 7:**
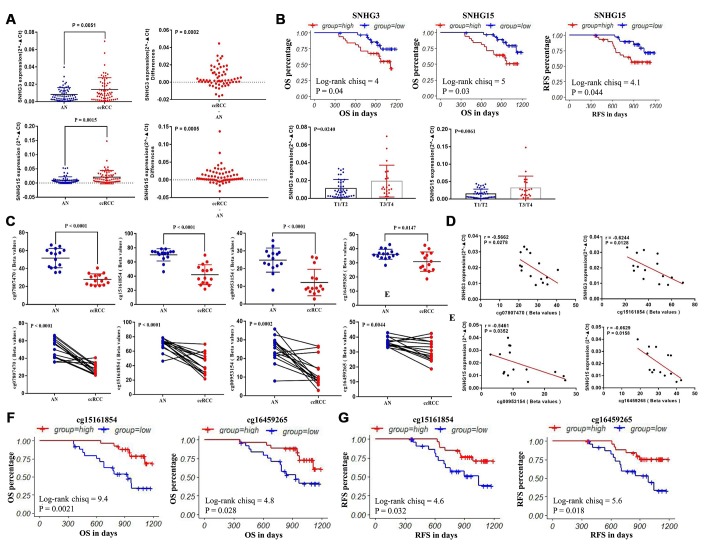
**The validation of the expression patterns and the methylation status of SNHG3 and SNHG15.** (**A**) q-RT-PCR analysis of SNHG3 and SNHG15 expression in ccRCC and adjacent normal renal tissues. (**B**) Patients were grouped according to the median cutoff of SNHG3 and SNHG15 expression for OS detection, patients were grouped according to the median cutoff of SNHG15 expression for RFS detection, and the correlation between SNHG3 and SNHG15 expression and the tumor stage of ccRCC patients. (**C**) Pyrosequencing analysis of cg07807470, cg15161854, cg00953154 and cg16459265 methylation levels between ccRCC and matched adjacent normal tissues. (**D**) The methylation levels of cg07807470 and cg15161854 were negatively associated with the expression of SNHG3. (**E**) The methylation levels of cg00953154 and cg16459265 were negatively associated with the expression of SNHG15. (**F**) Patients were grouped according to the median cutoff of cg15161854 and cg16459265 methylation status for OS detection. (**G**) Patients were grouped according to the median cutoff of cg15161854 and cg16459265 methylation status for RFS detection.

62 paired clinical samples were utilized to validate the expression patterns of SNHG3 and SNHG15, and 15 paired clinical samples were used to examine the DNA methylation status of 4 CpG sites (cg07807470, cg15161854, cg00953154, cg16459265) (Figure 1), and the clinical information of these 62 ccRCC patients are also showed in Table 6. q-RT-PCR was performed to confirm the expression of SNHG3 and SNHG15 in these 62 paired clinical samples. Consistent with the results from TCGA datasets, the expression levels of SNHG3 and SNHG15 were remarkably higher in ccRCC tissues than that of adjacent normal renal tissues (Figure 7A). Results of the log-rank test showed that high expression of SNHG3 and SNHG15 was associated with the shorter OS, and high expression of SNHG15 was also related to the shorter RFS. Moreover, the expression levels of SNHG3 and SNHG15 were significantly higher in advanced-stage ccRCC than in early-stage ccRCC (Figure 7B).

**Table 6:** The clinical characteristics of 62 ccRCC patients used for validation.

**Clinicopathologic characteristics**	**n (%)**
Age	
<60	33 (53.2)
>=60	29 (46.8)
Overall Survival	
Alive	42 (67.7)
Dead	20 (32.3)
Tumor size	
< 2cm	7 (11.3)
>= 2 cm, <5cm	40 (64.5)
>= 5cm	15 (24.2)
Gender	
Male	46 (74.2)
Female	16 (25.8)
Tumor stage	
T1/T2	41 (66.1)
T3/T4	21 (33.9)
Historical Grade	
G1/G2	39 (62.9)
G3/G4	23 (37.1)

In addition, the methylation status of 4 CpG sites (cg07807470, cg15161854, cg00953154 and cg16459265) in 15 paired clinical samples were tested through pyrosequencing. In the whole population, the methylation levels of 4 methylation sites were consistent with the TCGA cohort. The methylation levels of cg07807470, cg15161854, cg00953154 and cg16459265 were significantly lower in ccRCC compared with adjacent normal tissues (Figure 7C). The representative results of pyrosequencing for methylation status in two paired samples were shown in Supplementary Figure 1. Besides, the methylation levels of cg07807470 (r=-0.5662) and cg15161854 (r=-0.6244) were significantly negatively correlated with SNHG3 expression (Figure 7D), while the methylation levels of cg00953154 (r=-0.5461) and cg16459265 (r=-0.6629) were negatively associated with SNHG15 expression (Figure 7E), which were consistent with the results from the TCGA datasets. Furthermore, the methylation levels of cg15161854 with a higher correlation with SNHG3 expression and cg16459265 with a higher correlation with SNHG15 expression in another 36 available ccRCC samples were tested through pyrosequencing for survival analysis. Consistent with the survival analysis results of TCGA samples, our results showed that low methylation levels of cg15161854 and cg16459265 were associated with the shorter OS (Figure 7F) and RFS (Figure 7G) of ccRCC patients.

## DISCUSSION

Because nearly 1/3 of ccRCC patients have localized or distant metastasis at the initial diagnosis and almost all ccRCC show radiotherapy and chemotherapy resistance finally, the 5-year survival rates of ccRCC patients remains as low as 20% [6, 24, 25]. Therefore, finding new and effective prognostic biomarkers is critical for patients with ccRCC due to the disappointing clinical outcomes.

The past few years have seen the emergence of certain snoRNAs as potential regulators of cell fate, and insight into the molecular mechanisms by which snoRNAs may carry out these regulatory functions, in addition to the modification of rRNAs, has started to appear in the scientific literature [26]. Interestingly, the modification of ribosomal biogenesis was associated with the development of cancer, suggesting that the classical function of snoRNA may contribute to the development of cancer [27, 28]. In addition to the initial evidence of snoRNA involvement in cancer development, increasing evidence has demonstrated that dysregulated small nucleolar RNA host genes may contribute to multiple cancer progression. For example, SNHG1 was upregulated in human colorectal cancer tissues, and high SNHG1 expression was associated with shorter OS [29]; SNHG1 contributed to sorafenib resistance by activating the Akt pathway in hepatocellular carcinoma cells [30]; SNHG17 was upregulated in non-small-cell lung cancer (NSCLC), and the knockdown of SNHG17 inhibited the proliferation and migration and promoted the apoptosis of NSCLC cells [31]. However, there has been relatively little research on the clinical roles of small nucleolar RNA host genes in ccRCC.

In our study, we compared the expression patterns of 20 small nucleolar RNA host genes in ccRCC and adjacent normal tissues based on TCGA-KIRC data. Results showed that compared with adjacent normal tissues, SNHG1, GAS5, SNHG3-8, SNHG11, SNHG12, SNHG15-17, SNHG20, SNHG22 and SNHG25 were significantly upregulated in ccRCC tissues, while SNHG9, SNHG10, DANCR and SNHG14 were remarkably downregulated in ccRCC tissues. Moreover, after adjustment for confounding factors, increased SNHG3 expression was a potential risk factor of shorter OS, while upregulated SNHG15 expression was an independent predictor of shorter RFS in ccRCC. When concerning the relationship between these lncRNAs and the progression of ccRCC, SNHG3 and SNHG15 were closely related to some clinical parameters of ccRCC, such as tumor stage, histological grade and remote metastasis. In addition, we also performed q-RT-PCR to verify the expression of SNHG3 and SNHG15 with 62 paired clinical samples. Results of the log-rank test showed that high expression of SNHG3 and SNHG15 was associated with the shorter OS, and the expression levels of SNHG3 and SNHG15 were significantly higher in advanced-stage ccRCC than in early-stage ccRCC. Although survival data from both the validation samples and the TCGA samples showed that the prognosis of ccRCC patients with high expression of SNHG3 and SNHG15 was poor, the median survival time of validation patients was much shorter than that of TCGA patients (about 1000 days vs. 1800-2000 days). Clinically, approximately 30% of localized ccRCC patients will nevertheless develop recurrence or metastasis after surgical resection of their tumor [32]. At present, several prognostic factors for patients with recurrent RCC after a nephrectomy for localized disease were established. In a previous study, each RCC patient was given a total risk score of 0 to 5, with one point for each of five prognostic variables (recurrence < 12 months after nephrectomy, serum calcium > 10 mg/dL, hemoglobin < lower limit of normal, lactate dehydrogenase > 1.5x upper limit of normal, and Karnofsky performance status < 80%), and patients were categorized into low- (score = 0), intermediate- (score = 1 to 2), and high-risk subgroups (score = 3 to 5) [33]. The result showed that the median survival time for low-risk, intermediate-risk, and high-risk patients was 76, 25, and 6 months, respectively. Therefore, in addition to the above 5 factors, more studies are needed on the impact of other factors, such as living environment, underlying diseases and medical conditions, on the median survival time of RCC patients.

Consistent with our results, SNHG3 and SNHG15 have been reported to be significantly upregulated in several types of cancer. SNHG3 was overexpressed in colorectal cancer [34], ovarian cancer [35], osteosarcoma [36] and hepatocellular carcinoma [37], and its upregulation was associated with poor OS. Moreover, a recent study demonstrated that the higher expression of SNHG3 could predict worse clinical prognosis, and knockdown of SNHG3 could significantly inhibit the proliferation and metastasis of ccRCC *in vitro* and *in vivo* [38]. Similarly, SNHG15 was also overexpressed in colorectal cancer [22, 39], thyroid carcinoma [40] and osteosarcoma [41]. Besides, SNHG15 could promote cell proliferation, invasion and drug resistance in colorectal cancer, suggesting its potential as prognostic marker and target for RNA-based therapies [42]. Furthermore, SNHG15 was significantly upregulated in RCC tissues and cell lines compared with its adjacent normal tissues and a proximal tubule epithelial cell line, and SNHG15 knockdown could inhibit RCC proliferation, invasion and migration [43]. Taken together, all the results indicated that the highly expressed SNHG3 and SNHG15 played crucial roles in the occurrence and development of ccRCC.

Epigenetic alterations such as DNA methylation, histone modification, and loss of genome imprinting play crucial roles in the formation and progression of cancer [44]. Over the past decade, many researches have indicated the presence of abnormal DNA methylation in various types of tumor [45–47]. In addition, it is well known that abnormal DNA methylation includes global hypomethylation and regional hypermethylation, in which regional hypermethylation is usually associated with gene silencing. For example, hypermethylation of the CpG shore of the Shh gene resulted in Shh loss, and inhibition of DNA methylation increased Shh expression to halt the initiation of bladder cancer at the early stage of progression [48]; DNA methylation at an enhancer of the three prime repair exonuclease 2 gene (TREX2) was linked to decreased TREX2 gene expression and protein expression, which may affect drug-induced DNA damage repair in laryngeal cancer [49]; and Epigenetic Silencing of miRNA-338-5p and miRNA-421 drived SPINK1-Positive Prostate Cancer [50]. Besides, a recent study reported that downregulation of CLDN7 due to promoter hypermethylation contributed to human ccRCC progression and poor prognosis [51], indicating DNA methylation may also play vital roles in ccRCC. However, all of above studies have focused on the effect of DNA methylation on mRNA or miRNA, and the regulation of DNA methylation on lncRNA was rarely reported.

During our study, we examined the correlation between SNHG3, SNHG15, SNHG12 and SNHG17 expression and their DNA methylation status. Results showed that the expression levels of SNHG3 and SNHG15 were more likely to be modulated by methylation in ccRCC. The methylation levels of cg07807470 and cg15161854 were negatively associated with SNHG3 expression, and the methylation levels of cg00953154, cg03440944 and cg16459265 were negatively correlated with SNHG15 expression in ccRCC. Moreover, low methylation levels of cg07807470, cg15161854, cg00953154 and cg16459265 were significantly associated with poor OS of ccRCC patients. Low methylation levels of cg07807470, cg15161854 and cg16459265 were also related to the shorter RFS. In addition, we verified the methylation status of the 4 CpG sites and their correlation with their corresponding SNHGs with 15 paired clinical samples by pyrosequencing and q-RT-PCR. Furthermore, the methylation levels of cg15161854 with a higher correlation with SNHG3 expression and cg16459265 with a higher correlation with SNHG15 expression in another 36 available ccRCC samples were tested through pyrosequencing for survival analysis. Consistent with the survival analysis results of TCGA samples, our results showed that low methylation levels of cg15161854 and cg16459265 were associated with the shorter OS and RFS of ccRCC patients. Taken together, SNHG3 and SNHG15 expression levels might be substantially modulated by DNA methylation in ccRCC.

In summary, we for the first time comprehensively determined the clinical significance of small nucleolar RNA host genes and the effect of DNA methylation on their expression in ccRCC. Although, we identified that SNHG3 and SNHG15 may have great clinical value to act as diagnostic biomarkers and indicators to evaluate the survival and progression of ccRCC, the number of cases can still be improved. Currently, the mechanisms of SNHG3 and SNHG15 in regulating the prognosis of patients with ccRCC are still not fully understood. More experiments are needed to further validate the regulation of DNA methylation on SNHG3 and SNHG15 expression.

## MATERIALS AND METHODS

### Ethics statement

All procedures performed in studies involving human participants were in accordance with the ethical standards of the institutional and/or national research committee and with the 1964 Helsinki declaration and its later amendments or comparable ethical standards. This study was approved by the Biomedical Research Ethics Committee of Peking University First Hospital.

### TCGA datasets

Level-3 RNA-sequencing data, the clinicopathological and survival data of patients with ccRCC were downloaded from TCGA (https://portal.gdc.cancer.gov/). The data availability of the patients included were summarized in Figure 1. Briefly, 539 ccRCC and 72 adjacent normal renal tissues were included in this study. Their clinical and survival data, including tumor stage, lymph node, metastasis, pathological stage, neoplasm histologic grade, OS and RFS, were downloaded. In addition, DNA methylation data (measured by the Infinium Human- Methylation450 BeadChip) that contains the data of 325 ccRCC and 160 adjacent normal renal tissues were also downloaded.

### Clinical samples for validation

A total of 62 paired tissues samples (adjacent normal renal tissue and ccRCC) were collected from ccRCC patients who underwent radical or partial nephrectomy at Peking University First Hospital. All the samples were immediately snap frozen in liquid nitrogen for long-term preservation until RNA or DNA extraction. This study was approved by the Biomedical Research Ethics Committee of Peking University First Hospital (Beijing, China, IRB00001052-18004). Written informed consent was also obtained from all patients.

### Total RNA extraction and quantitative real-time PCR

Total RNA was extracted from the 124 tissue samples using the TRIzol reagent (Invitrogen; Thermo Fisher Scientific Inc.), according to the manufacturer’s instructions. cDNA was generated using reverse transcription (TansGEN, Beijing, China). q-RT-PCR was performed using the ABI PRISM 7000 Fluorescent Quantitative PCR System (Applied Biosystems, Foster City, CA, USA), according to the manufacturer’s instructions, and normalized to GAPDH. All experiments were repeated at least three times. The detailed primer sequences included in this study are shown in Supplementary Table 1.

### Pyrosequencing

Genomic DNA was extracted from 15 paired clinical samples using the QIAamp DNA FFPE Tissue kit (QIAGEN, Hilden, Germany). The concentration and purity of these DNA samples were determined with a spectrophotometer (NANO-DROP 2000, Thermo Scientific, Waltham, MA, USA). Bisulfite conversion of total 500 ng purified DNA in each sample was performed with QIAGEN 59124-EpiTect Plus DNA Bisufite Kit according to manufacturer’s instructions. The bisulfite conversed DNA was amplified with Kapa Biosystems Hs Polymerase With Dntps250u KK5516 (KAPA, USA) with reaction setup: 34.8ul PCR-grade water, 10ul 5x KAPA buffer, 1ul dNTP (10Mm/each), 1ul forward prime (50pM/ul), 1ul reverse primers (50pM/ul), 2ul bisulfite-treated DNA (25ng) and 0.2ul Taq (5U/ul) in total 50 μL each reaction and with following thermal cycle condition: initial denaturation at 95 °C for 3 mins, denaturation at 94 °C for 30 s, annealing at 50 °C for 30 s, extension at 72 °C for 1 min executed for 40 cycles followed by extension at 72 °C for 7 min and hold at 4 °C. The amplicons were then subjected to pyrosequencing with PyroMark Q96 ID (Qiagen, Hilden, Germany). All primers used are presented in Supplementary Table 1.

### Statistical analysis

Welch’s unequal variances t-test was used to detect differences in continuous variables. The Pearson’s correlation test was conducted to assess the correlations between lncRNAs and hypoxia. The extent of correlations between the expression of SNHG3, SNHG12 and SNHG17 and their CpG sites’ DNA methylation levels were also evaluated. The receiver operating characteristic (ROC) curve was used to assess the diagnostic effectiveness of these aberrantly expressed lncRNAs in ccRCC. The prognostic roles of lncRNAs were examined with the Kaplan–Meier method, and the log-rank test was conducted to determine the significance of the difference between the survival curves. The univariate and multivariate cox analyses of these lncRNAs were also performed. A P-value < 0.05 represented statistical significance. The statistical analyses were all carried out by R language and GraphPad Prism 7.00.

## Supplementary Material

Supplementary Figure 1

Supplementary Table 1
