# Supplementary material for: Discovery and validation of the prognostic value of the lncRNAs encoding snoRNAs in patients with clear cell renal cell carcinoma
Source: Aging (Albany NY). 2020 Mar 3;12(5):4424–44. doi: 10.18632/aging.102894 (PMC7093172; doi:10.18632/aging.102894)
Supplement: Supplementary Figure 1 [file aging-12-102894-s001..pdf]

SUPPLEMENTARY FIGURE

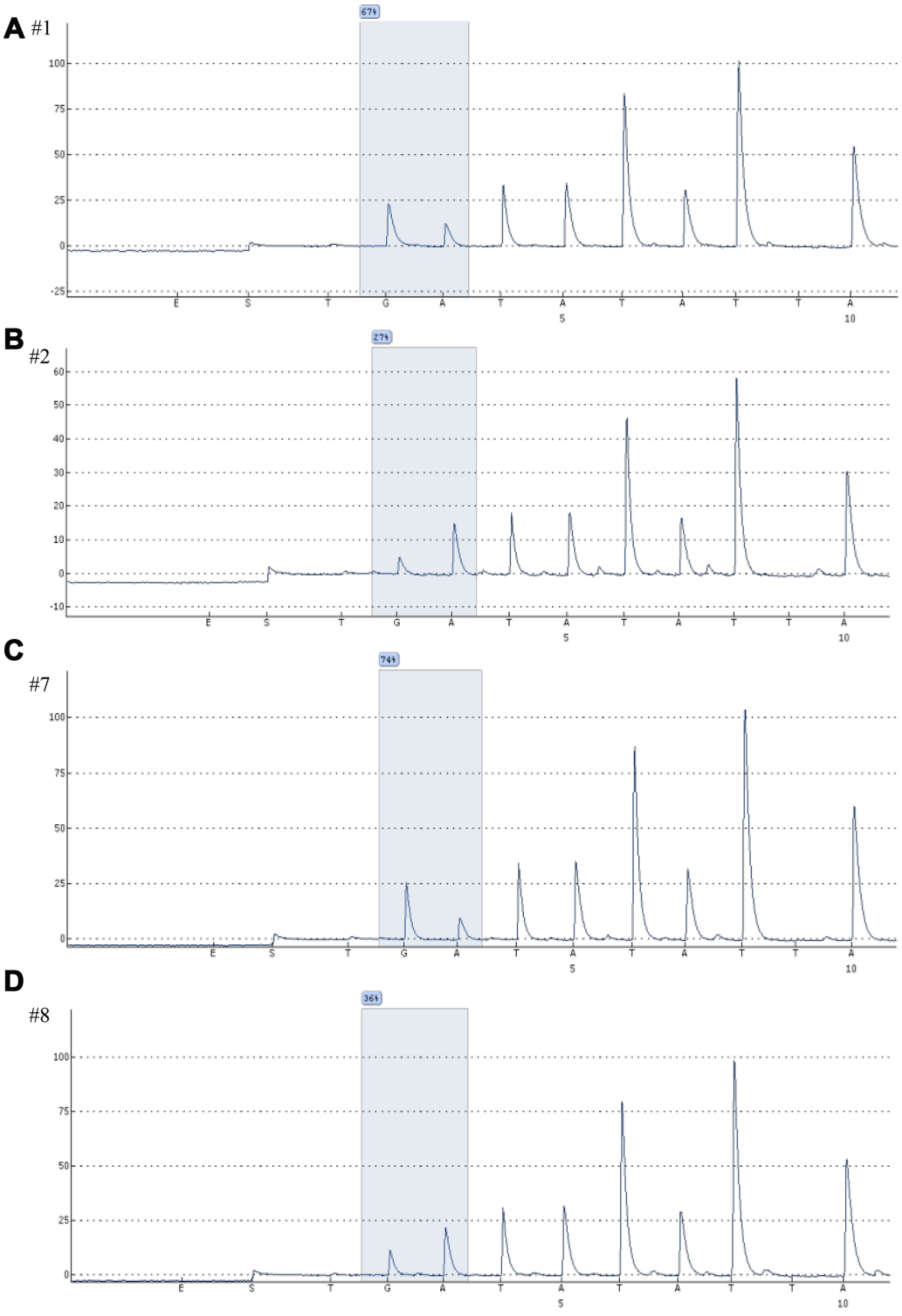

Supplementary Figure 1. The representative results of pyrosequencing for cg15161854 methylation level in two paired samples. Increased methylation shown in adjacent normal renal samples no. 1 (**A**) and no. 7 (**C**) and low methylation in ccRCC no. 2 (**B**) and no.8 (**D**).
