# Supplementary material for: Discovery and validation of the prognostic value of the lncRNAs encoding snoRNAs in patients with clear cell renal cell carcinoma
Source: Aging (Albany NY). 2020 Mar 3;12(5):4424–44. doi: 10.18632/aging.102894 (PMC7093172; doi:10.18632/aging.102894)
Supplement: Supplementary Table 1 [file aging-12-102894-s002..pdf]

SUPPLEMENTARY TABLE

Supplemental Table 1. The detailed primer sequences for q-RT-PCR and pyrosequencing.

**q-RT-PCR**				
Gene	Primer	Sequence (5′-3′)	Nt	Tm
SNHG3	Forward	GACGATCTGCACTTCGCATT	20	59
	Reverse	TGCTCCAAGTCTGCCAAAGAT	21	59.3
SNHG15	Forward	TGGCAGACCTGTACTCCGTA	20	59.96
	Reverse	GACTAGACTGCCGAAGACCG	20	59.9
GAPDH	Forward	GAAAGCCTGCCGGTGACTAA	20	60.32
	Reverse	GCATCACCCGGAGGAGAAAT	20	59.82

**pyrosequencing**				
CpG site	Primer	Sequence (5′-3′)	Length of amplicon	5' modification
cg07807470	Forward	GTAGAGTGGGATTTGAATATTTATTGATAG	276	5'-Biotin
	Reverse	AAAAACAAACATCAAATTCCCTACTC		
	Sequencing	AGATTTATGTAGTTATAGTAGGA		
cg15161854	Forward	TGGGATTATAGTTGTGAGTTATTTTGT	200	5'-Biotin
	Reverse	CCTTCAACAATACCATTCAAATTCTTAACT		
	Sequencing	AACCACAAACATACAATC		
cg00953154	Forward	TAGGTTTAGTTTTAGGAAGGTAGAGT	83	5'-Biotin
	Reverse	ACTATCACAACAACTCCTTTACA		
	Sequencing	GGTAGAGTTGGGGTT		
cg16459265	Forward	TTGGGTGAATGAGGGTAGT	197	5'-Biotin
	Reverse	ACACCAATCTATACAAAACCCAAAACAC		
	Sequencing	GGTGAATGAGGGTAGTA		
